# Feasibility of carbon‐ion radiotherapy for oral non‐squamous cell carcinomas

**DOI:** 10.1002/hed.25618

**Published:** 2019-01-24

**Authors:** Hiroaki Ikawa, Masashi Koto, Kazuhiko Hayashi, Morio Tonogi, Ryo Takagi, Takeshi Nomura, Hiroshi Tsuji, Tadashi Kamada

**Affiliations:** ^1^ Hospital of the National Institute of Radiological Sciences National Institutes for Quantum and Radiological Science and Technology Chiba Japan; ^2^ Department of Oral and Maxillofacial Surgery Nihon University School of Dentistry Tokyo Japan; ^3^ Department of Oral Pathobiological Science and Surgery Tokyo Dental College Tokyo Japan; ^4^ Department of Oral Medicine Oral and Maxillofacial Surgery, Tokyo Dental College Tokyo Japan

**Keywords:** carbon‐ion radiotherapy, particle therapy, head and neck malignancies, inoperable oral cancer, osteoradionecrosis

## Abstract

**Background:**

This study evaluated carbon‐ion radiotherapy (C‐ion RT) for oral non‐squamous cell carcinomas (non‐SCC).

**Methods:**

We retrospectively obtained data from 74 patients who underwent C‐ion RT for oral malignancies between April 1997 and March 2016. The C‐ion RT was administered in 16 fractions at a total dose of 57.6 or 64.0 Gy (relative biological effectiveness).

**Results:**

Forty‐three patients had salivary gland carcinomas, 29 patients had mucosal melanoma, and 2 patients had other types of pathologies. The tumors were classified as T1‐T3 (24 cases), T4a (21 cases), or T4b (29 cases). The median follow‐up was 49 months. The 5‐year rates were 78.8% for local control, 36.2% for progression‐free survival, and 58.3% for overall survival. Although 10 patients developed grade 3 osteoradionecrosis after C‐ion RT, all patients maintained their mastication and deglutition functions after sequestrectomy and prosthesis placement.

**Conclusion:**

C‐ion RT was effective for oral non‐SCC and had acceptable toxicities.

## 1 INTRODUCTION

The standard treatment for oral malignancies is surgery,^1^, ^2^, ^3^ and definitive radiotherapy (RT) is usually performed for inoperable cases. Squamous cell carcinoma (SCC) accounts for approximately 90% of all oral malignancies and is generally radiosensitive, although non‐squamous cell carcinoma (non‐SCC) cancers, which account for approximately 6%‐10% of all oral malignancies, are relatively radioresistant. Thus, RT has a limited role for non‐SCC cases,^4^, ^5^ which highlights the need for an effective treatment modality for patients with inoperable oral non‐SCC cancer.

Carbon ion RT (C‐ion RT) has a higher linear energy transfer and a greater relative biological effectiveness (RBE) than photon RT.^6^ Furthermore, various reports have described promising results from C‐ion RT for radioresistant tumors, such as salivary gland carcinoma (SGC) and mucosal melanoma of the head and neck.^7^, ^8^, ^9^ Koto et al.^8^ evaluated 46 patients with locally advanced parotid gland carcinoma who were followed‐up for a median of 62 months and reported 5‐year rates of 74.5% for local control and 70.1% for overall survival (OS). In addition, Koto et al.^9^ evaluated 260 patients with mucosal melanoma who underwent C‐ion RT and reported 5‐year rates of 72.3% for local control and 44.6% for OS. However, osteoradionecrosis (ORN) remains a major complication of C‐ion RT for head and neck malignancies, with grade ≥3 ORN developing in 4.8% of these cases.^10^ The relatively high risk of ORN in this setting is likely related to the proximity of the oral malignancy to the jawbone, relative to other head and neck malignancies. Nevertheless, few studies have evaluated C‐ion RT for oral malignancies and there are no reported data on related adverse events, including ORN. Therefore, the present study aimed to evaluate the safety and efficacy of C‐ion RT for oral non‐SCC.

## 2 PATIENTS AND METHODS

### 2.1 Patient and tumor characteristics

Between April 1997 and March 2016, 74 patients with oral non‐SCC cancer underwent C‐ion RT at our institution. Patient and tumor characteristics are summarized in Table 1. The eligibility criteria for the present study were patients who underwent definitive C‐ion RT for oral non‐SCC with: (1) a histologically confirmed malignant tumor, (2) a medically inoperable tumor or refusal of surgery, (3) age of 15‐80 years, (4) a Karnofsky performance status score of ≥60, (5) an N0‐N2b M0 classification, (6) a grossly measurable tumor, (7) no prior RT to the C‐ion RT‐treated area, and (8) no serious medical or psychological conditions precluding the safe administration of treatment.

**Table 1:** Patient and tumor characteristics

*Age, years*	No. of patients (%)
Median	56
Range	31‐79
*Sex*	
Male	37 (50)
Female	37 (50)
*Tumor status*	
Naïve	58 (78)
Recurrent	16 (22)
*Operability*	
Yes	47 (64)
No	27 (36)
*Tumor location*	
Hard palate	52 (70)
Alveolar ridge	9 (12)
Buccal mucosa	8 (11)
Anterior tongue	4 (5)
Floor of mouth	1 (1)
*Tumor classification*	
T1	1 (1)
T2	2 (3)
T3	21 (30)
T4a	21 (27)
T4b	29 (39)
*Node classification*	
N0	70 (95)
N1‐2b	4 (5)
*Skull base invasion*	
Yes	23 (31)
No	51 (69)
*Histology*	
Salivary grand carcinoma	
Adenoid cystic carcinoma	34 (46)
Adenocarcinoma	4 (5)
Epithelial myoepithelial carcinoma	2 (3)
Mucoepidermoid carcinoma	1 (1)
Acinic cell carcinoma	1 (1)
Carcinoma ex pleomorphic adenoma	1 (1)
Mucosal melanoma	29 (39)
Other	
Spindle cell carcinoma	2 (3)

Abbreviations: N, node; T, tumor.

Tumor locations were classified based on the International Classification of Diseases for Oncology, 3rd Edition (ICD‐O‐3) topography codes corresponding to the oral tongue, gums, floor of the mouth, hard palate, and cheek mucosa.^11^ All patients were restaged according to the seventh edition of the International Union Against Cancer TNM staging system.^12^

The study's retrospective protocol was approved by our institutional review board (17‐022), complied with the Declaration of Helsinki, and was registered in the UMIN database (UMIN000029522).

### 2.2 Carbon‐ion radiotherapy

The carbon‐ion doses were expressed as photon‐equivalent doses by multiplying the physical dose by the RBE of the carbon ions. The biological flatness of the spread‐out Bragg peak was normalized by the survival fraction of salivary gland tumor cells at the distal region of the spread‐out Bragg peak, where the RBE of carbon ions is estimated to be 3.0.^6^ The patients' treatment characteristics are shown in Table 2. The C‐ion RT was administered at doses of 57.6 or 64.0 Gy (RBE) in 16 fractions over 4 weeks (ie, 4 fractions per week). When a wide range of skin, mucosa, or jaw bone was included in the target volume (eg, for oral mucosal melanoma), the dose was set to 57.6 Gy (RBE) in 16 fractions over 4 weeks. Forty‐nine patients received a dose of 57.6 Gy (RBE) and 25 patients received a dose of 64.0 Gy (RBE).

**Table 2:** Treatment characteristics

*Dose and fraction, n (%)*	Value or no. of patients (%)
57.6 Gy (RBE)/16 fractions	49 (66)
64.0 Gy (RBE)/16 fractions	25 (34)
*Gross tumor volume*, cm^3^	
Median	33.85
Range	1.5‐186.1
*Planning target volume*, cm^3^	
Median	148.3
Range	22.7‐369.2

Abbreviations: RBE, relative biological effectiveness.

A custom‐made mouthpiece was used to maintain the positions of the maxillary bone, mandibular bone, and tongue.^13^ The patients were positioned in customized cradles (Moldcare; Alcare, Tokyo, Japan) and immobilized using a low‐temperature thermoplastic shell (Shellfitter; Kuraray, Osaka, Japan). A set of CT images with a slice interval of 2.5 mm was taken for treatment planning. During the treatment planning CT and irradiation, the patients were instructed to not swallow. Determination of the gross tumor volume (GTV) was based on contrast‐enhanced magnetic resonance (MR) images, CT images, and intraoral endoscopic examination findings. The clinical target volume (CTV) had minimum margins of 5.0 mm added around the GTV. In cases with adenoid cystic carcinoma, the CTV included the neural tracts to the skull base and peripheral site to account for any perineural spread. In cases with mucosal melanoma, the GTV including the melanosis was defined as the gross extent of the tumor, based on the intraoral endoscopic examination findings. The CTV included the entire anatomic sites where the tumors were located. A margin of 2‐3 mm was added around the CTV to create the planning target volume (PTV). The CTV margins were reduced as necessary near critical organs (eg, the mandibular bone, maxillary bone, eyeball, optic nerve, optic chiasm, and brain stem). The target reference point dose was defined as the isocenter, and an isodose line representing 90% of the reference point dose encompassed the PTV. In this study, 73 patients were treated with passive beam irradiation and 1 patient, who was the last patient included in this study, was treated with spot scanning beam irradiation. Three‐dimensional treatment planning was performed using HIPLAN software (National Institute of Radiological Sciences, Chiba, Japan)^14^ and Xio‐N (ELEKTA, Stockholm, Sweden; and Mitsubishi Electric, Tokyo, Japan). The dose calculation algorithm at our hospital was updated from HIPLAN to Xio‐N in 2013.

Nineteen of the 29 patients (66%) with mucosal melanoma received either concurrent or adjuvant chemotherapy, which included dimethyl traizeno imidazole carboxamide (DTIC). Patients with other tumors did not receive concomitant or adjuvant therapy involving surgery or chemotherapy. The treatment methods for locoregional recurrence and distant metastases were not restricted.

### 2.3 Evaluation and follow‐up examination

Follow‐up consisted of CT or MRI and endoscopic examinations every 2‐3 months for the first 2 years and every 3‐6 months thereafter. Local control was defined as no evidence of tumor regrowth in the PTV. Regional control was defined as no evidence of regional lymph node recurrence or oral cavity skip lesions outside the PTV. Acute and late reactions in normal tissues were classified according to version 3.0 of the National Cancer Institute's Common Terminology of Criteria for Adverse Effects.

### 2.4 Statistical analysis

Survival times were calculated from the first day of C‐ion RT. The cumulative incidences of local control, progression‐free survival (PFS), and OS were evaluated using the Kaplan–Meier method and log‐rank test. Variables with univariate *P*‐values of <.1 were included in a multivariate Cox proportional hazards model. The effect of prescribed doses on developing ORN was assessed with the Chi‐square test. Differences were considered statistically significant at two‐sided *P*‐values of <.05, and all analyses were performed using IBM SPSS software (version 19; IBM Corp., Armonk, New York).

## 3 RESULTS

The median follow‐up was 49 months (range: 9‐204 months) and all patients completed the planned C‐ion RT during a median treatment time of 28 days (range: 24‐31 days). None of the patients had a treatment time that was prolonged by >1 week. No patients were lost to follow‐up. Among the 14 patients with local recurrence, 13 patients developed recurrence within the PTV and 1 patient developed recurrence at the margin of the PTV. Of the 14 patients with local recurrence, 4 patients underwent salvage surgical therapy, 4 palliative care, 1 chemotherapy, 1 nivolumab, and 1 cyber knife treatment. The treatment was unknown for 3 patients.

Among the 14 patients who developed regional recurrence, 13 patients had cervical lymph node metastasis and 1 patient had a recurrent tumor in the masticatory space. Of the 13 patients with cervical lymph node metastasis, 8 patients received elective neck dissection, 2 received elective neck dissection with chemotherapy, 1 received elective neck dissection with chemoradiotherapy, 1 received cyber knife treatment, and 1 received palliative care.

Thirty patients developed distant metastasis. At the last follow‐up date, 34 patients had died because of their disease and 10 patients had died because of unrelated causes (pneumonia [*n* = 2], acute heart failure [*n* = 1], malignant lymphoma [*n* = 1], sepsis [*n* = 1], and unknown causes [*n* = 5]). Of the 5 patients who had died because of unknown causes, 4 patients had been diagnosed with local recurrence and 1 with secondary stomach cancer on the last day of observation.

**Figure 1:**
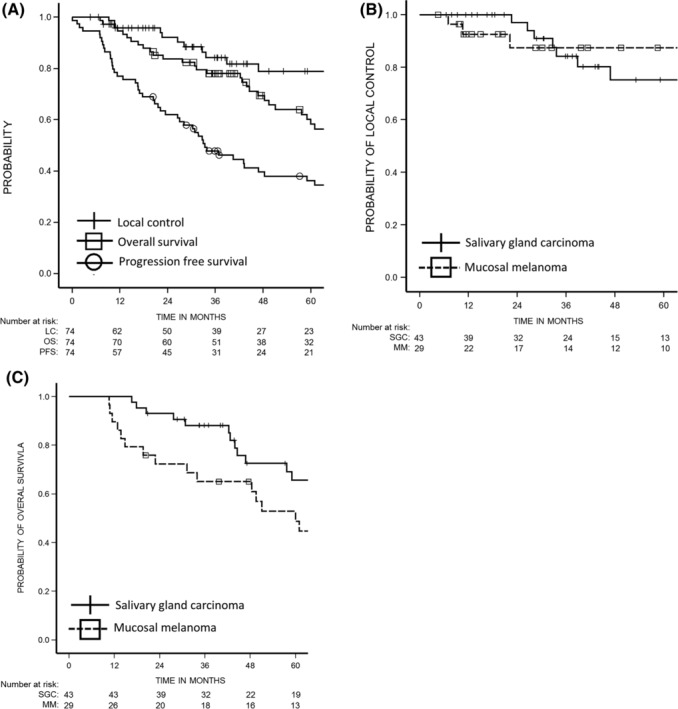
A, Rates of local control, progression‐free survival, and OS among all 74 patients. B, Local control rates according to histological subtype. The 5‐year local control rates among patients with SGC and mucosal melanoma were 75.2% and 87.4%, respectively. C, Overall survival rates according to histological subtype. The 5‐year overall survival rates among patients with SGC and mucosal melanoma were 65.7% and 48.8%, respectively

The Kaplan–Meier curves for local control, PFS, and OS are shown in Figure 1A. The cumulative 3‐ and 5‐year local control rates were 84.2% (95% confidence intervals [CI]: 74.5%‐93.8%) and 78.8% (95% CI: 67.3%‐90.4%), respectively. The 3‐ and 5‐year PFS rates among all patients were 47.8% (95% CI: 36.3%‐59.3%) and 36.2% (95% CI: 24.7%‐47.7%), respectively. The 3‐ and 5‐year OS rates among all patients were 78.0% (95% CI: 68.4%‐87.5%) and 58.3% (95% CI: 45.9%‐70.6%), respectively.

**Figure 2:**
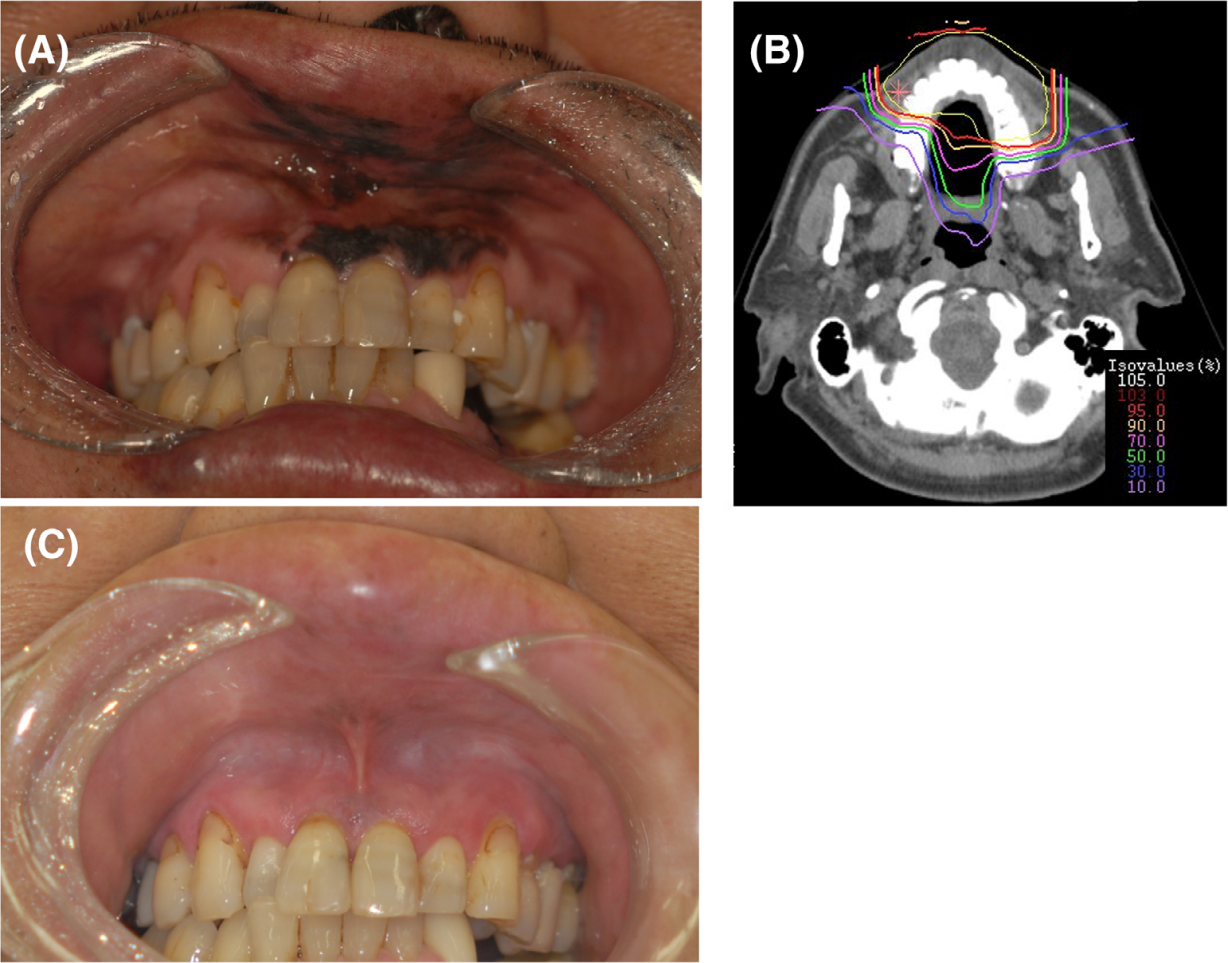
A 64‐year‐old male was seen with mucosal melanoma of the upper gingiva invading the maxillary bone. There was no lymph node swelling and no evidence of distant metastasis at presentation, and clinical stage was evaluated as T4aN0M0. The patient declined radical surgery and was referred for carbon ion radiation therapy (C‐ion RT). A, Intra‐oral endoscopic examination finding before C‐ion RT reveals mucosal melanoma in the upper gingiva. B, Dose distribution of C‐ion RT. C‐ion RT was administered at 57.6 Gy (RBE) in 16 fractions using 2 ports. The shown isodose lines correspond to 95%, 90%, 70%, 50%, 30%, and 10% dose areas. The planning target volume was demarcated by yellow lines. (C) Intra‐oral endoscopic examination finding 20 months after treatment shows that the tumor had completely disappeared. No grade ≥2 osteoradionecrosis was developed [Color figure can be viewed at wileyonlinelibrary.com]

The Kaplan–Meier curves for local control and OS according to histological subtype are shown in Figure 1B and Figure 1C. The 5‐year local control rates among 43 patients with SGC and 29 patients with mucosal melanoma were 75.2% (95% CI: 58.7%‐91.7%) and 87.4% (95% CI: 73.9%‐100.0%), respectively (Figure 1B). The 5‐year OS rates among 43 patients with SGC and 29 patients with mucosal melanoma were 65.7% (95% CI: 49.5%‐81.8%) and 48.8% (95% CI: 29.7%‐67.8%), respectively (Figure 1C). Figure 2A‐C shows a representative case of a patient with mucosal melanoma who was treated with C‐ion RT.

### 3.1 Prognostic factors

The results of the univariate and multivariate analyses of factors that might predict local control, PFS, and OS are shown in Table 3. None of the factors significantly predicted local control. The univariate analysis indicated that male sex was a significant risk factor for poor PFS (*P* = .012) and the multivariate analysis confirmed the independence of this association (hazard ratio [HR]: 1.969, 95% CI: 1.148‐3.377, *P* = .014). The univariate analyses revealed that OS was associated with age, sex, histology, and GTV. However, there was no significant difference in the multivariate analyses.

**Table 3:** Factors affecting local control, progression‐free survival, and overall survival

		Local control			Progression‐free survival			Overall survival		
		Univariate analysis	Multivariate analysis		Univariate analysis	Multivariate analysis		Univariate analysis	Multivariate analysis	
Factors	No. of patients	*P* value	*P* value	HR (95% CI)	*P* value	*P* value	HR (95% CI)	*P* value	*P* value	HR (95% CI)
*Age*										
<56 years	36	.310			.737			.021	.245	1.493 (0.760‐2.931)
≥56 years	38									
*Sex*										
Female	37	.139			.012	0.014	1.969 (1.148‐3.377)	.023	.151	1.602 (0.842‐3.048)
Male	37									
*Tumor status*										
Naïve	58	.549			.836			.860		
Recurrent	16
*Operability*										
Yes	47	.750			.785			.953		
No	27									
*Tumor location*										
Hard palate	52	.208			.830			.481		
Others	22
*T classification*										
T1‐3	24	.843			.888			.730		
T4a‐4b	50									
*N classification*										
N0	70	.286			.134			.321		
N1‐2b	4
*Skull base invasion*										
No	51	.198			.655			.979		
Yes	23									
*Histology*										
SGC	43	.160			.564			.054	.113	1.704 (0.883‐3.289)
MM	29									
Others	2									
*Gross tumor volume*										
<33.85 mL	37	.107			.672			.075	.099	1.728 (0.902‐3.311)
≥33.85 mL	37									
*Dose*										
64.0 Gy (RBE) /16 fr.	25	.157			.289			.789		
57.6 Gy (RBE) /16 fr.	49									

Abbreviations: CI, confidence interval; fr., fractions; HR, hazard ratio; MM, mucosal melanoma; T, tumor; N, node; RBE, relative biological effectiveness; SGC, salivary grand carcinoma.

### 3.2 Normal tissue reactions

Acute grade 2 and 3 mucosal reactions were observed in 21 patients and 43 patients, respectively. Grade 2 and 3 skin reactions were observed in 13 patients and 1 patient, respectively (Table 4). No grade ≥4 mucosal or skin reactions were observed. Late grade ≥3 reactions were observed in 22 patients, including grade 3 ORN (10 patients), and grade 4 optic nerve disorders (3 patients). No late grade 5 reactions were observed.

**Table 4:** Acute and late adverse reactions (grade ≥2)

Type of adverse reaction	Grade 2	Grade 3	Grade 4	Grade 5	Total
*Acute reactions*					
Mucositis	21	43	0	0	64
Dermatitis	13	1	0	0	14
*Late reactions*					
Osteoradionecrosis	27	10	0	0	37
Trismus	10	0	–	–	10
Dysphagia	6	0	0	0	6
Mucositis	5	1	0	0	6
Brain injury	5	0	0	0	5
Optic nerve disorder	1	0	3	–	4
Otitis media	0	3	0	0	3
Oral hemorrhage	1	1	0	0	2
Hearing impairment	1	1	0	–	2
Glaucoma	1	0	0	–	1
Epistaxis	1	0	0	0	1
Recurrent laryngeal nerve palsy	1	0	0	0	1
Facial nerve disorder	1	0	–	–	1
Soft tissue necrosis	0	1	0	0	1
Meningitis	0	1	0	0	1
Cataract	0	1	–	–	1

The 10 patients (13.5%) were diagnosed with grade 3 ORN based on severe pain and underwent sequestrectomy involving the maxilla (7 patients), the mandible (2 patients), or both the mandible and maxilla (1 patient). The median interval between the first date of irradiation and the detection of grade 3 ORN was 42.9 months (range: 3‐93 months). In addition, the median interval between the diagnosis of grade 1 ORN and the first intervention (grade 3 ORN) was 31.2 months (range: 1.9‐89.4 months). The incidences of grade >3 ORN were not significantly different (*P* = .244) between the two doses, with values of 10.2% (5/49 patients) in the low‐dose group and 20% (5/25 patients) in the high‐dose group. After the sequestrectomy, 3 patients underwent plate reconstruction of the mandible, 1 patient underwent bone graft transplantation (vascularized costal cartilage) for the maxilla, 2 patients received zygomatic implants, and 4 patients did not undergo additional interventions. Among the 4 patients who underwent bone reconstruction, 2 patients (1 patients with mandibular bone reconstruction and 1 patient with maxillary bone reconstruction) experienced failure with subsequent removal of the plate and transplanted bone, although the soft tissue graft was preserved. Thus, these 2 patients maintained their masticatory function. Among the 8 patients who developed maxillary defects after sequestrectomy, 6 patients maintained their mastication and deglutition functions after receiving maxillary obturator prostheses and 2 patients maintained their functions without maxillary obturator prostheses.

Three patients developed grade 4 ipsilateral blindness because of optic neuropathy. All 3 patients had tumors that had invaded the intracranial space and were close to the optic nerve. No ocular or visual toxicities were observed on the healthy sides.

## 4 DISCUSSION

Photon RT is one option for treating locally advanced oral malignancies,^2^, ^3^ although most non‐SCC lesions are considered radioresistant and are associated with poor clinical outcomes.^4^, ^5^ Thus, C‐ion RT has potential as a definitive treatment for radioresistant tumors. The present study revealed promising clinical outcomes and acceptable toxicities after C‐ion RT for oral non‐SCC, which suggests that it is feasible for patients with inoperable locally advanced oral non‐SCC.

One potentially critical complication of RT is ORN. Kuhnt et al. reported that 6.6% of 775 patients with head and neck cancer developed severe ORN that required extensive surgical intervention after photon RT, with the highest frequency being observed for oral cavity tumors (13.6%).^15^ Similarly, the present study revealed an incidence of 13.5% for grade 3 ORN among patients with oral malignancy who underwent C‐ion RT, which is higher than the incidence from our previous study of head and neck malignancies (4.8%).^10^ Thus, it appears that C‐ion RT and photon RT were associated with similar incidences of grade 3 ORN, despite the fact that the areas of ORN were localized because of the dose‐localization properties of carbon ions.^16^

Sequestrectomy is generally performed in cases with advanced ORN involving the maxilla but typically results in maxillary defects, which create significant rehabilitative issues related to mastication and deglutition. However, the majority of maxillary defects can be reconstructed with an uncomplicated obturator,^17^ with maxillary obturator prostheses being used to restore mastication and deglutition functions in those cases.^18^, ^19^ In the present study, 6 of 8 patients with maxillary defects after sequestrectomy had mastication and deglutition functions that were preserved using maxillary obturator prostheses.

Surgical resection of necrotic tissue and immediate reconstruction are routinely performed for patients with advanced ORN of the mandible.^20^ However, radiation exposure compromises the integrity of the recipient vessels and negatively affects free flap viability, with both preoperative and postoperative radiotherapy being associated with increased flap complication rates.^21^ Furthermore, plate reconstruction after RT is also associated with many late complications, and Seol et al. have reported a significant decrease in the success of plate reconstruction among patients who underwent postoperative RT.^22^ In the present study, plate failure occurred in 1 of 3 patients who underwent mandibular bone reconstruction, which suggests that plate‐based mandibular bone reconstruction may have limited effectiveness and should be carefully considered after C‐ion RT. Nevertheless, the patient who experienced reconstruction failure had successful soft tissue transplantation without any major complications and preserved masticatory and deglutitive functions (because of the soft‐tissue free flap and prosthesis). Baumann et al.^23^ also reported that complications were significantly more common with bone flaps than with soft‐tissue flaps among 63 patients who underwent free flap reconstruction for mandibular ORN. Hanasono et al. reported good clinical outcomes and similar times to functional outcomes (eg, oral intake, postoperative diet, mouth opening) for posterior mandibular reconstruction using either a vascularized bone flap or soft‐tissue free flap among 74 patients.^24^ Thus, it appears that reconstruction using only soft tissue flaps after sequestrectomy may be acceptable for patients who are treated using C‐ion RT. In addition, we have reported that the presence of teeth within the PTV and the volume of the maxillary bone receiving ≥50 Gy (RBE) are independent risk factors for ORN after C‐ion RT.^10^ Therefore, to reduce the risk of ORN, we routinely perform preemptive extraction of teeth with poor prognoses that are near the irradiation field. Moreover, we use a custom‐made mouthpiece with a spacer to reduce the area of the jawbone that is exposed to high‐dose irradiation.^25^

The standard treatment for intraoral minor SGC is surgery, which provides 5‐year rates of 73.8%‐83.1% for local control and 71.8%‐73% for survival.^26^, ^27^, ^28^ However, in locally advanced cases, surgical resection may be limited by critical adjacent structures. Thus, although SGC is known to be radioresistant, photon RT has become a widely used option as adjuvant or definitive treatment for intraoral minor SGC, despite limited data regarding the clinical outcomes. Yorozu et al. reported that the 5‐year local control and OS rates were 54% and 63%, respectively, among 12 patients with intraoral minor SGC who underwent photon RT, although 3 of the 12 patients underwent postoperative radiotherapy.^4^ In the present study, the 5‐year rates of local control and OS were 75.2% and 65.7%, respectively, among 43 patients with gross tumors, which indicates that C‐ion RT is a potentially definitive treatment for locally advanced intraoral SGC.

Mucosal melanoma is typically aggressive and is associated with a poor prognosis. Retrospective analysis of Surveillance, Epidemiology, and End Results data from 1973‐2012 indicates that the 5‐year OS rate is approximately 25% among patients with oral mucosal melanoma.^29^ Photon RT is an option as adjuvant or definitive treatment, although mucosal melanoma is also considered radioresistant, and Wushou et al. reported that the 3‐year OS rate was 0% among 21 patients with primary oral mucosal melanoma treated using RT.^5^ Interestingly, C‐ion RT appears to provide benefits in terms of local control and survival relative to photon RT, as we reported 5‐year rates of 89.4% for local control and 57.4% for OS among 19 patients who only received C‐ion RT for oral mucosal melanomas.^30^ The present study included 19 patients (66%) who received concurrent chemotherapy, including DTIC, which did not significantly improve prognosis based on 5‐year rates of 87.4% for local control and 48.8% for OS. Nevertheless, a retrospective multicenter study of C‐ion RT for head and neck mucosal melanoma revealed that a survival benefit was associated with concurrent chemotherapy, including DTIC.^9^ Moreover, complementary activity against melanoma has recently been reported for ipilimumab (a cytotoxic T‐lymphocyte‐associated antigen‐4 checkpoint inhibitor) and nivolumab (a programmed death‐1 checkpoint inhibitor),^31^, ^32^ and Karlsson et al. have reported that checkpoint inhibitors are more effective than immunotherapy or chemotherapy in terms of survival and tumor response among patients with stage III/IV unresectable cutaneous melanoma.^33^ Therefore, a combination of C‐ion RT and immunotherapy may improve treatment outcomes among patients with oral mucosal melanoma.

In this study, dose–response was not observed for local control, PFS, and OS on multivariate analysis. However, Kaplan–Meier curves revealed a trend for better 5‐year local control rate of 64 Gy (RBE) (85.2%), as compared to 57.6 Gy (RBE) (75.8%). Therefore, the use of 64 Gy (RBE) might be effective for local control of oral non‐SCC, although it should be considered that diverse histology was included in this study. The incidence of grade >3 ORN was higher in cases where 64 Gy (RBE) was used than in cases where 57.6 Gy (RBE) was used; however, no statistically significant difference was found (*P* = .244). Furthermore, even if Grade 3 ORN appeared, the area of ORN was localized because of the dose conformity of C‐ion RT. Consequently, it was possible to maintain the masticatory and deglutitive function by prosthesis and reconstruction after sequestrectomy, particularly for maxillary ORN. Thus, 64 Gy (RBE) might be recommended as the standard prescribed dose for oral non‐SCC, with the exception of cases in which the mandibular bone or the maxillary bone is extensively irradiated, as in palatal malignant melanoma. The volume of jawbone receiving more than 50 Gy (RBE) in 16 fractions was found to be a risk factor of ORN.^10^ The technological advancements made in recent years in scanning‐beam and gantry‐based treatment delivery reduce the volume of the jawbone that is exposed to high‐dose irradiation, which may decrease the risk of ORN.^34^, ^35^

C‐ion RT is a promising treatment option for inoperable oral non‐SCC. Since April 2018, the public health insurance system in Japan has covered C‐ion RT for head and neck malignancies, with the exception of oral, laryngeal, and pharyngeal SCC.

## 5 CONCLUSION

The present study revealed that C‐ion RT provided promising outcomes in cases of non‐SCC, especially for SGC and mucosal melanoma. However, ORN was a significant complication, despite being limited to localized areas, and the risks and benefits should be carefully considered for each patient, especially when the tumor is in close proximity to the mandibular bone. Nevertheless, the patients' masticatory and deglutitive functions could be preserved using maxillary prostheses and mandible reconstruction using soft tissue flaps. Given the inherent limitations of a single‐center retrospective study, further studies are needed to validate our findings, although all of our patients had been treated consecutively using an integrated C‐ion RT protocol.

## CONFLICT OF INTEREST

None declared.
